# Transcriptional Differences between Canine Cutaneous Epitheliotropic Lymphoma and Immune-Mediated Dermatoses

**DOI:** 10.3390/genes12020160

**Published:** 2021-01-25

**Authors:** Nadja Gerber, Magdalena A. T. Brunner, Vidhya Jagannathan, Tosso Leeb, Nora M. Gerhards, Monika M. Welle, Martina Dettwiler

**Affiliations:** 1 Institute of Animal Pathology, Vetsuisse Faculty, University of Bern, Länggassstrasse 122, 3001 Bern, Switzerland; gerber.na@bluewin.ch (N.G.); maggali.b@googlemail.com (M.A.T.B.); nora.gerhards@wur.nl (N.M.G.); monika.welle@vetsuisse.unibe.ch (M.M.W.); 2 Dermfocus, Vetsuisse Faculty, University of Bern, 3001 Bern, Switzerland; vidhya.jagannathan@vetsuisse.unibe.ch (V.J.); tosso.leeb@vetsuisse.unibe.ch (T.L.); 3 Grosstierpraxis Weibel + Werner, Oberdorfstrasse 15, 3438 Lauperswil, Switzerland; 4 Institute of Genetics, Vetsuisse Faculty, University of Bern, Bremgartenstrasse 109A, 3001 Bern, Switzerland; 5 Wageningen Bioveterinary Research, Wageningen University & Research, Houtribweg 39, 8221 RA Lelystad, The Netherlands

**Keywords:** *Canis lupus familiaris*, dog, skin disease, cutaneous T-cell lymphoma, lupus erythematosus, cytotoxic dermatitis, RNA sequencing, FFPE tissue, transcriptome

## Abstract

Canine cutaneous epitheliotropic T-cell lymphoma (CETL) and immune-mediated T-cell predominant dermatoses (IMD) share several clinical and histopathological features, but differ substantially in prognosis. The discrimination of ambiguous cases may be challenging, as diagnostic tests are limited and may prove equivocal. This study aimed to investigate transcriptional differences between CETL and IMD, as a basis for further research on discriminating diagnostic biomarkers. We performed 100bp single-end sequencing on RNA extracted from formalin-fixed and paraffin-embedded skin biopsies from dogs with CETL and IMD, respectively. DESeq2 was used for principal component analysis (PCA) and differential gene expression analysis. Genes with significantly different expression were analyzed for enriched pathways using two different tools. The expression of selected genes and their proteins was validated by RT-qPCR and immunohistochemistry. PCA demonstrated the distinct gene expression profiles of CETL and IMD. In total, 503 genes were upregulated, while 4986 were downregulated in CETL compared to IMD. RT-qPCR confirmed the sequencing results for 5/6 selected genes tested, while the protein expression detected by immunohistochemistry was not entirely consistent. Our study revealed transcriptional differences between canine CETL and IMD, with similarities to human cutaneous lymphoma. Differentially expressed genes are potential discriminatory markers, but require further validation on larger sample collections.

## 1. Introduction

Canine cutaneous epitheliotropic T-cell lymphoma (CETL) is a neoplastic skin disease of dogs, in which the neoplastic T lymphocytes have a specific tropism for the epithelium of haired skin and mucocutaneous junctions [1,2,3]. The etiology and pathogenesis of CETL are largely unclear [1,2]. T lymphocyte-driven immune-mediated cytotoxic dermatitis (IMD) is a reaction pattern, in which inflammatory T-cells target the keratinocytes of the skin and mucocutaneous junctions [1,4,5,6]. The IMD reaction pattern is recognized in various cutaneous forms of canine lupus erythematosus (LE). A unifying feature of the different cutaneous LE forms is the deposition of immunoglobulins and complement factors in the basement membrane zone, which initiates a cytotoxic T-cell reaction restricted to the basal cell layer [4]. Furthermore, IMD is seen in canine panepidermal cytotoxic dermatitis (PCD), in which cytotoxic T lymphocytes are triggered by exogenous agents (e.g., drugs, viral infection) to react against epidermal keratinocytes in all layers of the epidermis [1,5]. Other cases of canine IMD remain idiopathic [1,5].

Clinically, canine CETL and IMD share several features, in particular in the first months of the disease. Both may manifest with variably pruritic erythematous macules, papules, plaques, erosions, ulcerations, crusts, alopecia, scaling and depigmentation affecting haired skin, mucocutaneous junctions, footpads and mucous membranes [2,4,7,8,9]. CETL is a disease of middle-aged to old dogs with a mean age of onset of 10–11 years [2,8]. IMD can occur in dogs of all ages, but some forms predominantly affect middle-aged to old dogs (e.g., old-dog erythema multiforme; generalized discoid Lupus erythematosus) [1,4,9]. The most important clinical differences between CETL and IMD are the clinical course and the prognosis. In CETL, the progression of skin lesions and metastasis to lymph nodes is a common finding, even after treatment with chemotherapy and immunomodulatory drugs [2,7]. A large retrospective study comprising 148 cases undergoing different treatment modalities reported a median survival time of 264 days (range 5–2992 days) [8]. IMD have a better prognosis, since lesions frequently respond to immunomodulatory treatment or may even resolve spontaneously, particularly when the trigger can be removed [4,6].

The gold standard for the diagnosis of IMD and CETL is the histopathological examination of skin biopsies of affected dogs. In both diseases, T lymphocytes—neoplastic in CETL, reactive in IMD—infiltrate the epidermis/mucosal epithelium, the adnexal structures and the dermis to variable extents [1,2,4,5,6,7]. Around 25% of CETL cases show pathognomonic Pautrier’s microabscesses [2]. In IMD, the inflammation is centered either on the basal cell layer of the epidermis and hair follicle epithelium (“interface”), and is combined with a lichenoid inflammation in LE cases, or presents with transepithelial infiltration and keratinocyte apoptosis in all epidermal layers in PCD cases [1,4,5,6].

Early-stage CETL lesions with low to moderate numbers of well-differentiated and solely intraepithelial neoplastic lymphocytes may be difficult to discriminate from cell-rich IMD lesions in routine histopathology slides [1,6]. In assessing the biopsies submitted to our dermatopathology service, about 3–5% of the total of CETL and IMD cases have ambiguous histopathological features, requiring additional lymphocyte clonality testing by PCR (PARR). With this test, the lymphocytic infiltrate can be classified as neoplastic if monoclonal vs. inflammatory if polyclonal [10]. However, there is the risk of inconclusive results, as PARR is not sensitive enough to detect small numbers of monoclonal lymphocytes in subtle CETL lesions, in particular when whole-biopsy paraffin sections are used, and/or a polyclonal background caused by secondary inflammation obscures the monoclonal signal [10]. Conversely, clonal expansion may occur in reactive inflammatory processes [10]. Supplemental immunohistochemical analyses (IHC) are of limited value, as the infiltrate consists mostly of cytotoxic T lymphocytes in both disease types [4,7,9]. Discriminatory immunohistochemical markers as used in human medicine either do not work on canine formalin-fixed and paraffin-embedded (FFPE) tissues, are not sensitive enough or are completely lacking [7,11]. In such equivocal cases, a reliable diagnosis is not possible at the time of biopsy analysis. As a consequence, only the clinical course and a possible follow-up biopsy will result in a definite diagnosis, with the risk of inappropriate treatment and impaired disease outcome in the time of diagnostic uncertainty.

For the discovery of markers reliably discriminating ambiguous CETL and IMD cases, the knowledge of molecular differences between neoplastic and inflammatory processes is crucial, but currently not sufficient. To expand the state of knowledge, our study aimed to investigate the transcriptional differences between canine CETL and IMD, as a basis for the further identification of candidate biomarkers discriminating between neoplasia and inflammation. We hypothesized that the transcriptome profiles of CETL and IMD biopsy samples are significantly different, and single differentially expressed genes and/or their proteins might serve as discriminating biomarkers. Encouraged by previous studies, in which comparative gene expression analyses have been performed on canine FFPE samples [12,13], we addressed our aims by global mRNA sequencing using archival FFPE biopsy samples from dogs afflicted by either CETL or IMD.

## 2. Materials and Methods

*Ethics statement:* All samples used in this retrospective study were archival biopsy samples taken and submitted to our institution for diagnostic purposes. By submission, the owner gave their consent for the future use of the tissues for research purposes. This procedure complies with the institutional ethical requirements, and obviates the requirement of animal experiment permission.

*Study samples and disease characterization:* Twenty formalin-fixed and paraffin-embedded (FFPE) biopsy samples from dogs with the histological diagnosis of immune-mediated dermatitis (IMD) (n = 11) and cutaneous epitheliotropic T-cell lymphoma (CETL) (n = 9) were selected from our institutional 2013–2017 archive. Of the eleven IMD cases, four were clinically and histopathologically compatible with lupus erythematosus (LE), and seven with panepidermal cytotoxic dermatitis (PCD). Inclusion criteria for all cases were the presence of a mild intraepithelial lymphocytic infiltrate with only minimal dermal involvement, but nevertheless allowing a histopathological diagnosis, and the availability of at least three punch biopsies from haired skin. Anamnesis and clinical follow-up data were retrieved from the referring veterinarians using a questionnaire.

From all FFPE blocks 3 µm sections were mounted on glass slides and stained with hematoxylin and eosin (H&E) and the periodic acid–Schiff reaction (PAS) according to routine protocols. The sections were evaluated blindly by one author, a board-certified veterinary pathologist, by assessing several histological parameters as listed in the supporting material (Table S2). Fungal infections were excluded in all cases using the PAS reaction.

To confirm the classification into the neoplastic and the inflammatory groups, respectively, a PCR for antigen receptor rearrangement (PARR) was performed. For this purpose, genomic DNA was extracted from the FFPE material using seven 16 µm sections each according to manufacturer’s instructions (QIAamp DNA FFPE Tissue Kit, QIAGEN, Hombrechtikon, Switzerland) with the exception that tissue lysis with proteinase K was performed overnight. DNA was quantified using the NanoDrop 1000 Spectrophotometer (NanoDrop Technologies, Wilmington, NC, USA). The amplification of the TCRγ locus was performed on a 96-well VeritiTM Thermal Cycler (Applied Biosystems, Rotkreuz, Switzerland) in duplicate as previously described [14]. The PCR product was visualized on the Fragment AnalyzerTM Automated CE System (Agilent, Basel, Switzerland) using the dsDNA 905 Reagent Kit, 1–500 bp (Agilent, Basel, Switzerland) according to manufacturer’s instructions. Two cases had a negative PARR result (no amplified sequences), but had clear-cut histopathological lesions and a compatible clinical disease course, enabling a reliable disease classification.

*RNA extraction:* Total RNA was extracted from 10 to 16 µm sections (15–30 per sample) of the FFPE tissue using the RNeasy FFPE Kit (QIAGEN, Hombrechtikon, Switzerland) according to manufacturer’s instructions. Residual genomic DNA was removed from the total RNA with a DNase treatment step according to the manufacturer’s protocol. The quantity and quality of the extracted RNA was analyzed using a NanoDrop 1000 Spectrophotometer (NanoDrop Technologies, Wilmington, NC, USA). RNA integrity (RIN) was assessed on a Fragment AnalyzerTM Automated CE System (Agilent, Basel, Switzerland). RNA samples were aliquoted and stored at −80 °C until further use.

*RNA Sequencing:* From the twenty cases, five cases with RIN values < 2.5 (range of all cases: 1.2–10.0), and/or with a Nanodrop A260/280 < 1.85 ratio (range: 1.79–2.03), were excluded, resulting in fifteen samples (CETL n = 6, PCD n = 6, LE n = 3) subjected to RNA sequencing (RNA-seq). Library preparation was performed without further RNA fragmentation with the SMARTer Stranded Total RNA-Seq Kit v2—Pico Input Mammalian, according to manufacturer’s instructions (Takara Bio, Saint-Germain-en-Laye, France). This protocol includes a ribosomal RNA depletion by ZapR v2 in the presence of mammalian-specific R-Probes. Subsequently, the libraries were sequenced on two lanes with 100 bp single-end sequencing cycles on an Illumina HiSeq 3000 (Illumina, Zurich, Switzerland).

*Raw data analysis:* The Illumina BCL output files with base cells were converted into the FASTQ format and demultiplexed. Data are available from the European Nucleotide Archive (accession no. PRJEB31872). Raw reads were filtered for low quality sequences and trimmed from adapters using the FastQC version 0.11.7 [15]. All reads that passed quality control were aligned to the dog reference genome CanFam3.1 using STAR aligner version 2.6.0c [16]. Reads were aligned using the following parameters: --outFilterType BySJout; --outFilterMultimapNmax 50; --alignSJoverhangMin 1; --outFilterMismatchNmax 2; --outFilterMismatchNoverLmax 0.04; --alignIntronMin 20; --alignIntronMax 1,000,000; --alignMatesGapMax 1,000,000. Aligned reads were counted using HTseq version 0.9.1 [17], and a NCBI transcript database (version 104) derived from the CanFam3.1 dog genome assembly. Quality control, mapping and feature counting were performed on UBELIX (http://www.id.unibe.ch/hpc), the HPC cluster at the University of Bern, and the necessary tools were loaded from Vital-IT.

**Figure 1:**
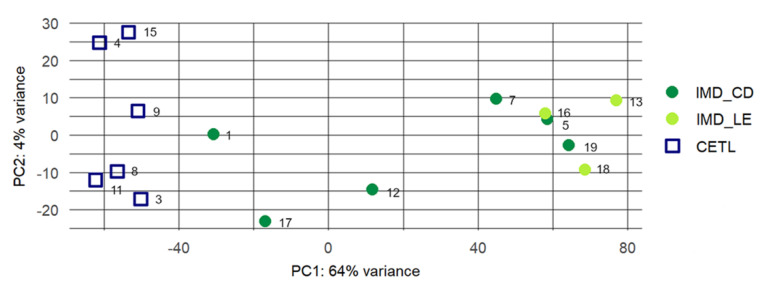
Principal component analysis of the RNA sequencing output. The PCA plot is calculated on the basis of 5000 genes most differentially expressed between canine CETL and IMD skin samples. Distances between samples reflect differences in the transcriptome profile of the samples.

*Differential expression analysis:* The DESeq2 package was used to read the HTseq count data [18]. A filtering step for low/non-expressed (counts 0/1) genes was done. For visualization, a principal component analysis (PCA) was performed with the logarithm transformed count data. Because there was no separation between PCD and LE cases in the PCA plot (Figure 1), we decided to perform the following experiments and analyses with only two groups, CETL and IMD, respectively, without the further subgrouping of the IMD cases. We also used Cook’s distance calculation with default DESeq2 function. Cook’s distance is a measure of how much a sample is influencing the fitted coefficients for a gene, and a large value of Cook’s distance indicates an outlier count. Subsequent differential gene expression analysis was performed with DESeq2 version 1.6.3 using the IMD group as control. DESeq2 applies a generalized linear model (GLM) to count data assuming a negative binomial distribution. Transcripts were considered differentially expressed with a Benjamini and Hochberg false discovery rate (FDR) of <0.01 (log2 fold-change (log2FC) <−0.58 and >0.58; *p* < 0.05).

*Pathway and gene enrichment analysis:* To look for enriched pathways and gene networks among the differentially expressed genes, we applied two different enrichment analysis tools, Ingenuity Pathway Analyis (IPA^®^, QIAGEN, Hombrechtikon, Switzerland) and KOBAS 3.0 [19]. The latter uses pathways from the KEGG database, and gene ontologies from the GO database. Because of the asymmetric distribution of the DESeq output in terms of gene numbers and log2FC between up- and downregulated genes, we performed two separate enrichment analyses, one including all genes with a logFC >1.5 (log2FC > 0.58; *p* < 0.05), and one including all genes with a logFC < −1.5 (log2FC < −0.58; *p* < 0.05), respectively, in the CETL group, as described previously [20].

*RT-qPCR:* In order to validate the RNA-seq results, and to assess the value of certain genes as a discriminating marker, RT-qPCR was performed using RNA from the 15 sequenced samples. We selected five candidate genes upregulated in the CETL group (*CD5*, *IL2RB*, *ILK*, *ITGB7* and *TCF7*) that have already been described to play a role in human cutaneous T-cell lymphomas and other forms of cancer [21,22,23,24,25,26,27,28]. Although not differentially expressed in the RNA-seq experiment, FOXP3, a transcription factor of regulatory T-cells, was selected for RT-qPCR due to its previously described association with several human skin diseases, including mycosis fungoides (MF) [29,30,31,32]. Extracted total RNA (1–2 µg per sample) was reverse transcribed using the GoScript™ Reverse Transcription Mix with Random Primers (Promega Corporation, Dübendorf, Switzerland), according to manufacturer’s specifications. Primers for genes of interest (*CD5*, *FOXP3*, *IL2RB*, *ILK*, *ITGB7*, *TCF7*) were designed using Primer3Plus [33]. Primers for reference genes (*RPL8*, *RPS19*) were chosen among seven genes previously described to be stably expressed in canine skin (*GUSβ*, *CZZ1*, *RPL8*, *RPL32*, *RPS5*, *RPS18*, *RPS19*) using the GeNorm algorithm [12,34,35,36,37]. Primer sequences are listed in Table 1.

Each primer pair was tested in a preliminary RT-qPCR assay for efficiency using a standard curve of six serial dilutions of a balanced mixture of cDNA from all samples. A negative control lacking any cDNA sample was included for each primer pair standard curve validation. Primer efficiencies ranged from 94 to 109%. Preliminary and definite RT-qPCR assays were performed with a 10 ng cDNA template in a 15 µl reaction using Fast Start Universal SYBR Green Master (Rox) (Roche Diagnostics, Rotkreuz, Switzerland). Each reaction was run in triplicate in three independent runs on a 7500 Fast Real-Time PCR System (Applied Biosystems, Rotkreuz, Switzerland) with the following conditions: 10 min 95 °C for activation, 40 cycles of 10 s 95 °C and 30 s 58 °C for amplification, completed by a dissociation cycle of 15 s 95 °C, 30 s 60 °C and 15 s 95 °C. Data were collected and processed with the 7500 Software v2.3. Standard curve and ΔΔCT-analysis (Pfaffl method) was performed in Excel. Differences in relative mRNA expression between CETL and IMD cases were calculated for each gene with a Man–Whitney-U test using GraphPad Prism 6.00 for Windows (GraphPad Software, LaJolla, CA, USA).

*Immunohistochemistry (IHC):* For the validation of candidate markers on the protein level, IHC for TCF7 and FOXP3 was performed on all samples submitted to gene expression analysis and the remaining samples with insufficient RNA quality (n = 20). IHC validation was limited to these two proteins due to a lack of antibodies for the other proteins working on canine FFPE tissue.

From all cases 3 µm tissue sections were mounted on glass slides, deparaffinized and rehydrated. Antigen retrieval was performed in citrate buffer (pH 6.0) for 20 min in a pressure cooker (107 °C, 3 bar). Nonspecific antibody binding was blocked by incubation with 10% normal goat serum in phosphate buffered saline (PBS) for 30 min, followed by primary antibody incubation (rabbit monoclonal anti-TCF7, diluted 1:100 in PBS (C.725.7, ThermoFisher Scientific, Reinach, Switzerland) and rat monoclonal anti-FOXP3, diluted 1:100 in PBS (FJK-16s, eBioscence, San Diego, CA, USA)) overnight at 4 °C. Endogenous peroxidase activity was blocked by incubation in 3% H_2_O_2_ for 15 min. After incubation for 30 min at room temperature (RT) with the pre-diluted biotinylated goat anti-mouse IgG secondary antibody (Agilent Technologies AG, Basel, Switzerland) and biotinylated goat anti-rat IgG (Vector laboratories, Burlingame, CA, USA), respectively, streptavidin-biotin-peroxidase (Agilent Technologies AG, Basel, Switzerland) was applied for 30 min at RT. The reaction was visualized by AEC substrate-chromogen (Agilent Technologies AG, Basel, Switzerland) application for 10 min. Counterstaining was performed using hematoxylin. A canine lymph node from our pathology archive served as positive control. As negative control, the primary antibody was replaced by monoclonal rabbit IgG serum (ab172730, abcam, Cambridge, UK) and monoclonal rat IgG2a kappa isotype control (eBR2a, eBioscence, San Diego, CA, USA), respectively, in equal dilutions. The specificity of the FOXP3 antibody in canine tissue has been shown previously [38], and the specificity of the TCF7 antibody was confirmed by western blot in the course of this study (Appendix A).

In addition, CD3 immunostaining using the LN10 clone (Leica Biosystems, Muttenz, Switzerland) in a 1:100 dilution on 3 µm tissue sections was performed on the BOND-III system (Leica Biosystems, Muttenz, Switzerland), complying with manufacturer’s instructions.

For evaluation, ten photomicrographs each of epidermis, dermis and adnexal structures in 40× magnification were taken from every stained section with a ProgRes^®^C5 microscope camera (Jenoptik, Jena, Germany). Labeled cells within the epidermis, dermis, adnexa, periadnexal tissue were counted manually using the MultiPoint tool of the ImageJ program 2018 [39]. The mean percentage of TCF7-positive and FOXP3-positive T-cells was calculated by dividing the number of CD3-positive cells by the number of TCF7-positive or FOXP3-positive cells, respectively, each counted in ten photomicrographs. This percentage was calculated for the whole skin biopsy, for epidermis and adnexa combined (“epithelium”), and for dermis and periadnexal tissue combined (“dermis”). The mean percentage was compared between CETL and IMD cases using a Man–Whitney-U test. Statistical analyses were performed with NCSS 12 Statistical Software (2018) (NCSS, LLC. Kaysville, UT, USA, http://www.ncss.com/software/ncss).

## 3. Results

### 3.1. Study Cohort

Signalment, overall survival time and the results of the PARR of the 20 cases are summarized in Table 2. Among the nine dogs with CETL, three were Cocker Spaniels, while other breeds were not overrepresented in both disease groups. The mean age at the time of diagnosis was 10 years 4 months (7 y 7 m to 13 y 9 m) in the CETL group, and 7 years 7 months (2 y 3 m to 11 y 8 m) in the IMD group. The median overall survival time of CETL patients was 166 (6–277) days. All CETL patients were euthanized due to the worsening of the skin lesions. At the end of the study, 6 out of 11 IMD cases were still alive, with either cured or therapeutically controlled disease (241–1086 days after diagnosis). One dog with IMD died after 1324 days due to reasons unrelated to the skin disease. Four panepidermal cytotoxic dermatitis (PCD) cases and one lupus erythematosus (LE) case with severe exfoliation required early euthanasia due to the severity of skin lesions (2–78 days after diagnosis). More details on clinical and histopathological findings are available in the Supplementary Materials (Tables S1 and S2).

### 3.2. RNA Sequencing and Raw Data Analysis

Single-end sequencing of the fifteen RNA libraries produced a mean number of 30 million (M) reads per sample on average (range: 25–36 M). The mean percentage of reads uniquely mapped to the genome was 82.6%, ranging from 75.5 to 86.9%. Among those, 29.7% on average mapped to the annotated canine transcriptome (range: 21.6–39.1%), resulting in 7 M counts per sample on average (Table S3).

### 3.3. Differential Gene Expression Analysis

**Figure 2:**
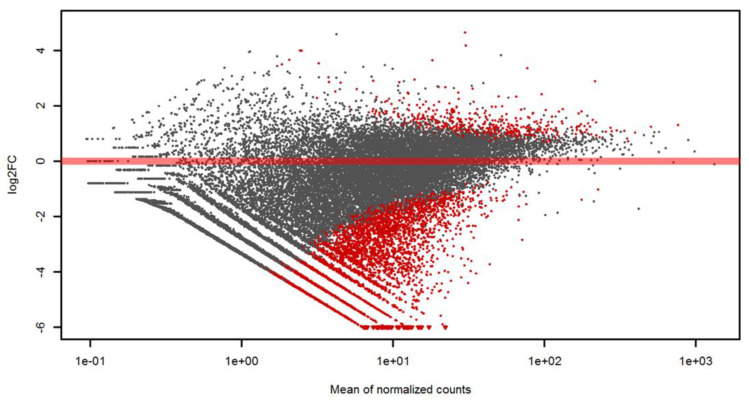
MA plot of the RNA sequencing output. The plot visualizes the differences in gene expression in a log2 ratio (log2FC) between the two groups. Each dot represents a gene; red dots indicate differential expression (FDR < 0.01).

The principal component analysis of the differentially expressed genes illustrates a clear separation of CETL and IMD cases with respect to gene expression (Figure 1). Six of the nine IMD cases clustered together, while three IMD cases were more distantly located. Cook’s distance test showed no outliers or any sample that was highly influential (Figure S1). As there was clustering of LE and PCD cases in this PCA, and because histologically, ambiguous CETL lesions may be akin to both LE and PCD lesions, we decided to use all IMD cases as one group for the subsequent analyses. Differential gene expression analysis resulted in 5489 differentially expressed genes (Table S4). Among those, 503 genes were upregulated, while 4986 genes were downregulated in CETL samples compared to IMD samples. The related MA plot is shown in Figure 2.

### 3.4. Pathway and Gene Enrichment Analysis

Enrichment analysis applied on the 503 genes with significantly higher expression in CETL samples resulted in 28 enriched IPA pathways, 1 KEGG pathway and 83 GO terms (*p* < 0.05) (Table S5). When all 4986 genes with significantly lower expression in CETL samples were used as input, 55 IPA pathways, 5 KEGG pathways and 61 GO terms were identified (*p* < 0.05) (Table S6). The top three enriched pathways and GO terms, respectively, for all three databases used are listed in Table 3 and Table 4. Information on genes involved in these pathways and gene networks is available in the supporting material (Tables S5 and S6).

### 3.5. RT-qPCR

**Figure 3:**
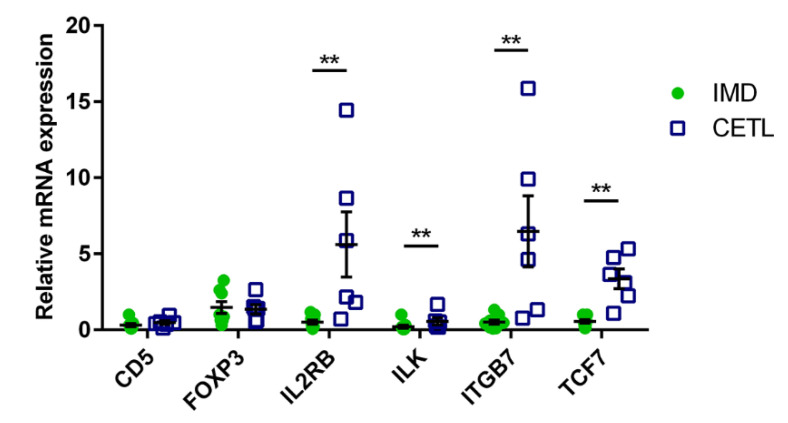
Relative mRNA expression of selected genes resulting from RT-qPCR. IMD cases depicted as green dots, CETL cases as blue squares; whiskers indicate the standard deviation of the mean; ** *p* < 0.05. The outliers in the *IL2RB* and *ITGB7* measurements are cases no. 3 and 8, and cases no. 8 and 11, respectively.

For five out of the six selected genes, the relative mRNA expression levels identified by RT-qPCR of the CETL and IMD samples were compatible with the RNA-seq results (Figure 3). *TCF7*, *ITGB7*, *ILK* and *IL2RB* were significantly upregulated (*p* < 0.05). Consistent as well with the RNA-seq results, *FOXP3* mRNA levels were not significantly different in the RT-qPCR (*p* = 0.99). Differences in *CD5* expression detected in the RNA-seq, however, could not be verified in RT-qPCR (*p* = 0.11). No significant difference in the mRNA levels was detected between the PCD and LE cases for all six genes tested (Figure S2).

### 3.6. Immunohistochemistry

**Figure 4:**
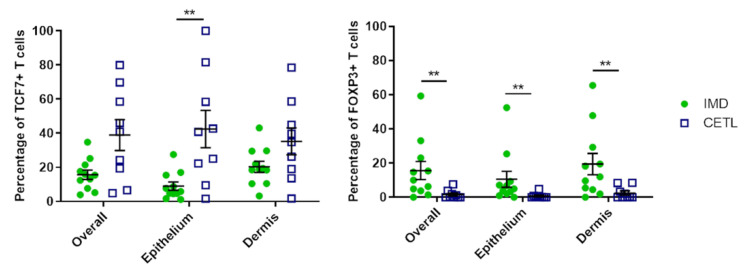
Results of the immunohistochemistry. Percentage of TCF7- and FOXP3-positive T-cells in the whole skin (overall), in the epithelial structures (epithelium) and the dermal portion (dermis); whiskers indicate the standard deviation of the mean; ** *p* < 0.05.

Staining with TCF7 and FOXP3 antibodies resulted in a clear nuclear signal in the T-cell zone of the control lymph node (Figure S3). In the study samples, the percentage of TCF7-positive T-cells was higher in the CETL group than in the IMD group, with the most significant differences in the epithelial compartment (Figure 4). In contrast, the percentage of FOXP3-postive T-cells was significantly lower in the CETL group (Figure 4).

## 4. Discussion

Here, we provide, to our knowledge, the first genome-wide RNA sequencing study comparing the transcriptome of canine CETL and IMD using archival lesional skin samples. As expected, T-cell neoplasia and T-cell-driven inflammation have different gene expression profiles, as shown by the principal component analysis. Interestingly, a distinct clustering among the IMD cases, namely panepidermal cytotoxic dermatitis (PCD) vs. lupus erythematosus (LE) cases, was not visible, which is most likely due to a certain pathogenetic heterogeneity among these cases, and is potentially influenced by the diverse anatomic origin and cellular composition of the biopsy samples. We validated the difference in the expressions of selected genes on the mRNA and protein levels, and could confirm significant differences between CETL and IMD, but not between PCD and LE cases. Thus, we performed differential gene expression analysis and pathway analyses only by comparing the major diseases, the CETL and the IMD group, respectively.

For the pathway analysis, we used two different enrichment analysis tools based on three different databases (KEGG, GO, IPA) in order to have an internal validation. We performed the enrichment analysis separately for up- and downregulated genes in CETL, as it has been shown that this approach can identify more relevant pathways, some of which might be missed in an overall enrichment analysis [20].

Among genes upregulated in CETL, the top enriched pathways and GO terms in all analyses included genes related to ribosomes and protein translation (eIF2 signaling, ribosome, ribosomal subunits), and genes related to cell–matrix interactions (ILK signaling, integrin signaling, focal adhesion). Ribosomal protein upregulation is described in several types of cancer [40,41,42,43,44]. However, the underlying mechanism is largely unknown due to the lack of functional analyses [41,43,44]. It remains open whether the upregulation is a prerequisite or a consequence of neoplastic transformation. Ribosomal biogenesis is also enriched in proliferating cells without having an active impact on carcinogenesis [45]. In contrast to many other cancer cells, T lymphocytes in CETL are usually not highly proliferative, as shown by Fontaine et al. using Ki67 as a proliferation marker [2]. The mitotic activity in our CETL cases was also low (Table S2), and was only slightly higher than in the IMD group. As such, the proliferation is unlikely to be the sole explanation for the higher ribosomal protein expression. Upregulated ribosomal biogenesis might therefore be related to the neoplastic transformation of T lymphocytes.

The second molecular process enriched in CETL was the interaction between cells and extracellular matrix (focal adhesion, ILK signaling and integrin signaling), encompassing the mechanical connection as well as the signaling [46]. This molecular process includes a number of integrin subunits, which are linked to several downstream cascades involved in cell proliferation, differentiation, apoptosis and migration [47]. Dysregulated integrin activity may contribute to tumor progression and metastasis formation [48]. A recent study on canine melanocytic tumors described integrins to be important upstream regulators favoring melanoma development [12].

Among genes with significantly lower expression in CETL, and which are thus upregulated in IMD, the enriched pathways included transmembrane receptor signaling in conjunction with G-protein coupled receptors. This protein family with over 800 members is the major class of sensory receptors, including receptors for chemokines, other inflammatory mediators and neuroactive substances, and is important for the migration of inflammatory and neoplastic immune cells [49,50]. In canine CETL, an upregulation of *CCR4* and *CCR10* expression compared to normal skin has been described [51]. In our study, *CCR4* and other chemokine receptors had a significant lower expression in the CETL group, which may be explained by the fact that *CCR4* and *CCR10* expression is increased also under inflammatory conditions [49]. Here, the inclusion of normal canine skin into our analyses would have improved the comparability of our results with those of previous studies.

On the single gene level, we found 83 differentially expressed genes described previously in gene expression analyses of human CETL (Table S7). In contrast, we could not find similarities to previous studies on canine CETL, although those veterinary studies focused only on a selection of chemokines, chemokine receptors, cytokines and markers for cytotoxicity analyzed by RT-qPCR. Moreover, they compared CETL to healthy skin and blood, respectively, and not to inflammatory disease, explaining, at least partially, the discrepancy [51,52]. In awareness of the differences between human and canine CETL, we selected four genes with reported upregulations in human CETL, *TCF7, IL2RB, CD5,* and *ITGB7* for validation on the mRNA level by RT-qPCR. We focused on genes with higher expressions in CETL, as our secondary aim was to identify potential biomarkers reliably and easily excluding a T-cell-driven inflammatory process.

Transcription factor 7 (TCF7), also known as T-cell factor 1 (TCF1), is an essential transcription factor of the WNT-signaling pathway. It is predominantly expressed in T lymphocytes and is important for the T-cell development [53]. The *TCF7* gene upregulation was described in some of the uncommon human mycosis fungoides (MF) cases as exhibiting an aggressive behavior [24]. Additionally, the protein expression of TCF7 was shown in human peripheral T-cell lymphomas expressing markers of Th1 activation [54]. By RT-qPCR, we could verify significantly increased mRNA levels in the CETL group, and we detected a higher percentage of TCF7-positive cells in the CETL group using IHC.

*IL2RB* is the gene encoding the interleukin-2 receptor (IL-2R) β subunit. Interleukin 2 (IL-2) and its receptor are critically involved in the control of T-cell growth [55]. IL-2Rα, another subunit of the high affinity receptor, is known to be upregulated in activated T-cells and in human Sézary syndrome, the leukemic form of MF [56,57]. While the expression levels of IL-2Rβ and IL-2Rγ are lower in resting T-cells, IL-2Rβ expression in particular is upregulated after T-cell activation [58]. In our study, *IL2RB* mRNA expression was upregulated in the CETL group in both RNA sequencing and confirmatory RT-qPCR. This upregulation might reflect a similar aberrant T-cell activation in canine CETL to that shown in the human disease.

*ITGB7* was selected because of its involvement in the enriched focal adhesion pathway. Moreover, the protein encoded by this gene forms dimers with integrin αEβ7, which is a described marker for epitheliotropism in human MF [59]. Our RT-qPCR results confirmed the significantly higher expression of *ITGB7* in the CETL group discovered by RNA sequencing.

*CD5* encodes the T-cell glycogen receptor CD5, the loss of which is a criterion for the diagnosis of a neoplastic process in early human MF [11]. Similar findings were described for canine CETL using immunohistochemistry on cryosections [7]. In our study, *CD5* expression was increased in the CETL group according to our RNA sequencing, which is consistent with the results from human studies [25]. However, RT-qPCR analysis did not confirm an increased *CD5* mRNA expression in CETL cases. Investigations on protein expression by IHC were not possible due to the lack of a functional antibody on canine FFPE tissues [7].

In addition to these four genes selected due to their overlap with human CETL studies, we selected *ILK* and *FOXP3* for validation. *ILK* encodes the integrin-linked kinase, the key protein of the upregulated ILK signaling pathway. ILK is a downstream enzyme in integrin signaling, with further connections to the WNT pathway, thus playing a role in cell adhesion, survival and cell cycle regulation [60]. Besides being upregulated in several human epithelial cancer types, ILK has been reported as a survival factor in human acute myelogenous leukemia, and as an important factor for T-cell trafficking and survival [26,27,28,61]. The increased *ILK* expression detected by RNA sequencing could be confirmed by RT-qPCR.

Although not significantly differentially expressed in our RNA-seq analysis, we investigated the expression of *FOXP3*, a transcription factor expressed in regulatory T-cells, by RT-qPCR and IHC. We selected *FOXP3*, because regulatory T-cells were recently described to play a role in several human skin diseases including MF [29,30,31,32]. The RT-qPCR results accorded with the RNA sequencing. Surprisingly, immunohistochemistry revealed a significantly decreased percentage of FOXP3-positive T-cells in the CETL group. In human MF, a reduced number of FOXP3-positive T-cells is associated with worse prognosis [30,32]. Our findings would be in line with this human situation, as canine CETL has a poor prognosis in most cases.

A limitation of this study is the use of FFPE tissue for RNA extraction, as formalin fixation leads to nucleic acid crosslinking and RNA fragmentation [62]. In order to yield the best possible RNA quality and quantity, we used an RNA extraction kit specifically designed for FFPE tissue and a library preparation kit suitable for highly degraded RNA, as specified by the manufacturers. Our transcriptome mapping percentage of 29.7% on average is compatible with yields from previous RNA-seq studies using FFPE tissue samples, and may be explained by the high amounts of conserved nuclear unspliced RNA due to formalin fixation [13,62,63]. These studies nevertheless all confirmed that RNA sequencing from FFPE tissue is possible and can produce biologically meaningful results. Moreover, as the confirmatory diagnosis in veterinary medicine is routinely made by histopathology on FFPE material, and the challenge of ambiguity between CETL and IMD occurs with this sample type, it is reasonable to use this material for biomarker screening by RNA-seq. The RNA-seq data quality in our study can be regarded as appropriate since our PCA plot appears adequate and gene expression differences could be confirmed by RT-qPCR. Nevertheless, as low exonic read mapping may hinder the detection of genes with low expression levels, one should consider increasing the sequencing depth when using FFPE tissue samples for RNA-seq [62].

For the discovery of diagnostic biomarkers reliably discriminating ambiguous CETL and IMD cases, the examination of a higher number of cases is needed. Furthermore, additional limitations are based on the retrospective character of the study. First, most dogs included had been treated with glucocorticoids and/or antibiotics. This may have influenced the transcriptional profile of the biopsies. Second, the investigated lesions may represent different stages (especially CETL cases), which may have an effect on the transcriptional profiles. Third, biopsies originated from various anatomical locations, and no site-matched non-lesional skin was included in the study, neither from dogs in the study cohort, nor from additional control dogs. Site-matched samples could have helped in filtering out site-specific background noise and potential differences in the gene expression profiles. Non-lesional control tissue was unfortunately not considered during the study design. Later on, the separate sequencing of normal skin samples would potentially have resulted in major batch effects, making comparison with the initially sequenced samples difficult to impossible [64]. A second sequencing experiment with additional normal skin samples was unfortunately not possible, as there was not enough material of the primary sample cases.

Despite these limitations, our study nevertheless provides new insights into the molecular differences between CETL and IMD, and is thus a first unbiased and comprehensive screening for potential biomarkers. Our DESeq dataset comprises hundreds of genes expressed differentially between CETL and IMD cases, which may be used for future investigations into mRNA and/or protein expression differences in a larger archival sample collection, or in a prospective study using fresh-frozen canine skin samples.

## Figures and Tables

**Table 1:** Primer sequences for quantitative real-time qPCR.

Gene	Accession	Forward	Reverse	Product Size (bp)
*CCZ1*	XM_536878.6	GCAGGAAGGGATTCTCCAG	GGTCCAGTAAGAAATCTTCCATAA	74
*GUS* *β*	NM_001003191.1	GTGCTGGATCAGAAACGCAA	CTTTGGGTTGTCTCTGGCGA	136
*RPL8*	XM_532360	CCATGAATCCTGTGGAGC	GTAGAGGGTTTGCCGATG	64
*RPL32*	XM_848016	TGGTTACAGGAGCAACAAGAAA	GCACATCAGCAGCACTTCA	100
*RPS5*	XM_533568	TCACTGGTGAGAACCCCCT	CCTGATTCACACGGCGTAG	141
*RPS18*	XM_532106	TGCTCATGTGGTATTGAGGAA	TCTTATACTGGCGTGGATTCTG	116
*RPS19*	XM_533657	CCTTCCTCAAAAAGTCTGGG	GTTCTCATCGTAGGGAGCAAG	95
*CD5*	XM_022405839.1	CTTAGGCTGGCCTTGAAGCT	ACACTGGTGTTGCAGTTGGA	143
*FOXP3*	NM_001168461.1	AAATTCCACAACATGCGCCC	AGGCAAACATGCGTGTGAAC	124
*IL2RB*	NM_001286851.1	TCCTGTGAGCTGCTCCCTAT	ATCCTCCACCTCTCCCCTTC	137
*ILK*	XM_022407778.1	CACGGTTAGGGGAGTGTGTC	CCGTGTGGCAAGTGACAAAG	163
*ITGB7*	XM_022411473.1	GACTCCAGCAACGTGGTACA	CCCTCTTCTCAGGATCCCCA	136
*TCF7*	XM_022425289.1	GCAGAGACTTTTCCCCGACA	GCATGAGCAGATTGAAGGCG	116

**Table 2:** Signalment, overall survival and PARR results of the study cohort.

Case No.	Disease Type (with Precise IMD Diagnosis)	Breed	Sex ^1^	Age at Time of Diagnosis (Years and Months)	Overall Survival Time (Days)	PARR ^2^	Tests Applied
1	IMD (PCD)	Jack Russel Terrier	M	10 y 10 m	78	polyclonal	RNA-seq, RT-qPCR, IHC
2	CETL	Mixed	F	7 y 9 m	166	clonal	IHC
3	CETL	Cocker Spaniel	MC	7 y 7 m	230	clonal	RNA-seq, RT-qPCR, IHC
4	CETL	Pyrenean Shepherd	FS	12 y	277	clonal	RNA-seq, RT-qPCR, IHC
5	IMD (PCD)	Briard	M	10 y 2 m	1324	polyclonal	RNA-seq, RT-qPCR, IHC
6	CETL	West Highland White Terrier	FS	11 y 8 m	187	negative	IHC
7	IMD (PCD)	American Staffordshire Terrier	MC	11 y 8 m	54	polyclonal	RNA-seq, RT-qPCR, IHC
8	CETL	Cocker Spaniel	FS	13 y 9 m	179	clonal	RNA-seq, RT-qPCR, IHC
9	CETL	Mixed	MC	11 y 3 m	18	clonal	RNA-seq, RT-qPCR, IHC
10	CETL	Cocker Spaniel	FS	11 y 1 m	6	clonal	IHC
11	CETL	Golden Retriever	F	9 y 2 m	57	clonal	RNA-seq, RT-qPCR, IHC
12	IMD (PCD)	Shetland Sheepdog	F	10 y 7 m	alive	polyclonal	RNA-seq, RT-qPCR, IHC
13	IMD (LE)	Border Collie	MC	5 y 3 m	alive	negative	RNA-seq, RT-qPCR, IHC
14	IMD (PCD)	Podenco Canario	FS	9 y 8 m	45	polyclonal	IHC
15	CETL	Boxer	M	8 y 8 m	134	clonal	RNA-seq, RT-qPCR, IHC
16	IMD (LE)	Magyar Vizsla	F	2 y 3 m	2	polyclonal	RNA-seq, RT-qPCR, IHC
17	IMD (PCD)	Great Pyrenees	FS	5 y 11 m	alive	polyclonal	RNA-seq, RT-qPCR, IHC
18	IMD (LE)	Rhodesian Ridgeback	M	2 y 6 m	alive	polyclonal	RNA-seq, RT-qPCR, IHC
19	IMD (PCD)	Yorkshire Terrier	FS	11 y 2 m	alive	polyclonal	RNA-seq, RT-qPCR, IHC
20	IMD (LE)	Tervuren	M	2 y 3 m	alive	polyclonal	IHC

^1^ M, male; MC, male castrated; F, female; FS, female spayed. ^2^ Negative, no amplicon detected.

**Table 3:** Top three enriched pathways and GO terms, respectively, among genes with significantly higher expression in CETL samples resulting from enrichment analyses with KOBAS 3.0 and IPA.

Pathway	Enrichment Analysis Tool	Database	*p*-Value
EIF2 signaling	IPA	IPA	1.66 × 10^−7^
ILK signaling	IPA	IPA	1.02 × 10^−6^
Integrin signaling	IPA	IPA	9.55 × 10^−5^
Ribosome	KOBAS	KEGG	1.87 × 10^−6^
Cytosolic ribosome	KOBAS	GO	1.87 × 10^−6^
Ribosomal subunit	KOBAS	GO	1.87 × 10^−6^
Focal adhesion	KOBAS	GO	1.87 × 10^−6^

**Table 4:** Top three enriched pathways and GO terms, respectively, among genes with significantly lower expression in CETL samples resulting from enrichment analyses with KOBAS 3.0 and IPA.

Pathway	Enrichment Analysis Tool	Database	*p*-Value
GABA receptor signaling	IPA	IPA	5.01 × 10^−12^
Cellular effects of sildenafil (Viagra)	IPA	IPA	8.51 × 10^−9^
Glutamate receptor signaling	IPA	IPA	8.51 × 10^−8^
Olfactory transduction	KOBAS	KEGG	2.01 × 10^−43^
Neuroactive ligand–receptor interaction	KOBAS	KEGG	2.70 × 10^−5^
Nicotine addiction	KOBAS	KEGG	1.39 × 10^−3^
Transmembrane signaling receptor activity	KOBAS	GO	2.86 × 10^−9^
Signaling receptor activity	KOBAS	GO	2.86 × 10^−9^
G-protein coupled receptor activity	KOBAS	GO	2.86 × 10^−9^

**Figure A1:**
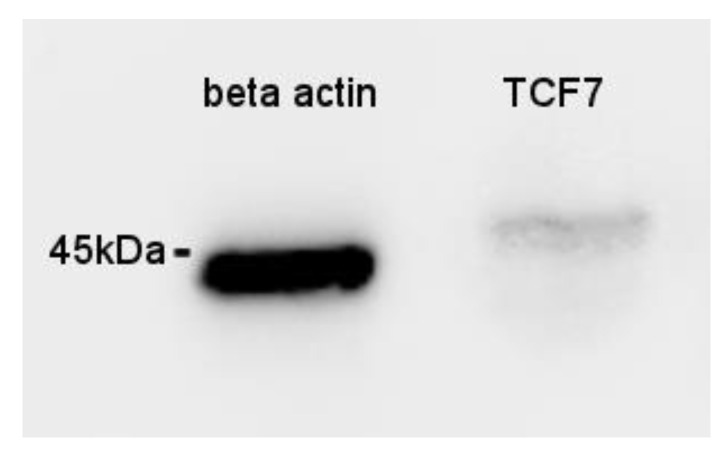
Western blot analysis confirming the specific binding of canine TCF7 by the antibody used.

## Data Availability

The data presented in this study are openly available from the European Nucleotide Archive at accession no. PRJEB31872.

## Supplementary Materials

The following are available online at https://www.mdpi.com/2073-4425/12/2/160/s1, Table S1. Table 2 extended with clinical parameters of the study population, Table S2. Histopathological parameters of the study population, Table S3. Summary of the raw data analysis results of the samples submitted to RNA-seq, Table S4. Results of the differential gene expression analysis, Table S5. List of enriched pathways and GO terms, respectively, among genes with significantly higher expression in CETL samples resulting from enrichment analyses with KOBAS 3.0 and IPA, Table S6. List of enriched pathways and GO terms, respectively, among genes with significantly lower expression in CETL samples resulting from enrichment analyses with KOBAS 3.0 and IPA, Table S7. Differentially expressed genes in our dataset previously described in human cutaneous epitheliotropic lymphoma, Figure S1. Boxplot depicting the Cook’s distances of genes across samples. Horizontal lines in the boxes represent the median (below 1 in all cases), boxes include the interquartile range, and whiskers delimitate the whole range of values calculated per sample, Figure S2. RT-qPCR (a) and immunohistochemistry (b,c) results with LE, PCD and CETL in three different groups, Figure S3. Positive control lymph nodes used for immunohistochemistry.

## Appendix A

Western blot analysis showing cross-reactivity of the TCF7 antibody with the canine orthologue.

*Materials and Methods:* In total, 90mg fresh frozen canine lymph node tissue was lysed in 1ml RIPA lysis buffer (50 mM Tris/HCl pH 7.4; 1% NP-40; 0.5% Na-deoxycholate; 0.1% SDS; 150 mM NaCl, 2 nM EDTA, 50 mM NaF) containing complete protease inhibitor cocktail (Roche, F. Hoffmann-La Roche Ltd., Basel, Switzerland) for 30 min on ice. It was homogenized using TissueLyser (QIAGEN, Hombrechtikon, Switzerland) for 5 min and cells were sonicated using Q700 Sonicator (Qsonica L.L.C, Newtown, CT, USA). After a centrifugation step (12,000rpm for 20 min at 4 °C), the protein concentration of the lysate was assessed using Pierce BCA assay kit (ThermoFisher Scientific, Reinach, Basel). A 2X SDS sample buffer was added, then the lysate was boiled for 10 min at 95 °C. Then 50µg total protein was loaded on a 12% SDS-PAGE and transferred to a PVDF membrane (Immobilon-P PVDF Membrane, MERCK, Zug, Switzerland) for 1h at 100 V. The membrane was blocked with 5% milk in TBS-T (100 mM Tris, pH 7.5, 0.9% NaCl, 0.05% Tween-20) for 1 h at room temperature and incubated with rabbit monoclonal TCF7 antibody (1:1000 dilution in 5% skimmed milk, C.725.7, ThermoFisher Scientific, Reinach, Switzerland) overnight at 4 °C. The membrane was rinsed twice with TBS-T and incubated with a horseradish peroxidase-conjugated polyclonal goat anti-rabbit antibody (1:2500, Cell Signaling Technologies, Leiden, Netherlands) in 5% skimmed milk for 1h at room temperature. After 4 washing steps with TBS-T, the bound complex was visualized using the chemiluminescent detection reagent (Amersham ECL Western blotting Detection Reagent, GE Healthcare Europe GmbH, Glattbrugg, Switzerland) on the Fusion FX (Vilber, Marne-la-Vallée, France). As a control for the protein content an additional blot was performed in parallel using β-actin primary antibody (A5441, dilution 1:2000, MERCK, Zug, Switzerland) and goat anti-mouse secondary antibody (1:2500, Cell Signaling Technologies, Leiden, The Netherlands).

*Results:* The western blot with the β-actin antibody showed a clear band slightly below 45 kDa, indicating the expected molecular weight. The TCF7 antibody produced a band that was weaker but also clear between 45 and 55kDa, which is slightly above the expected molecular weight of 42 kDa. This result demonstrates a specific cross-reaction of the TCF7 antibody with the canine protein orthologue (Figure A1).
