# Supplementary figures and images for: Transcriptional Differences between Canine Cutaneous Epitheliotropic Lymphoma and Immune-Mediated Dermatoses
Source: Genes (Basel). 2021 Jan 25;12(2):160. doi: 10.3390/genes12020160 (PMC7912288; doi:10.3390/genes12020160)

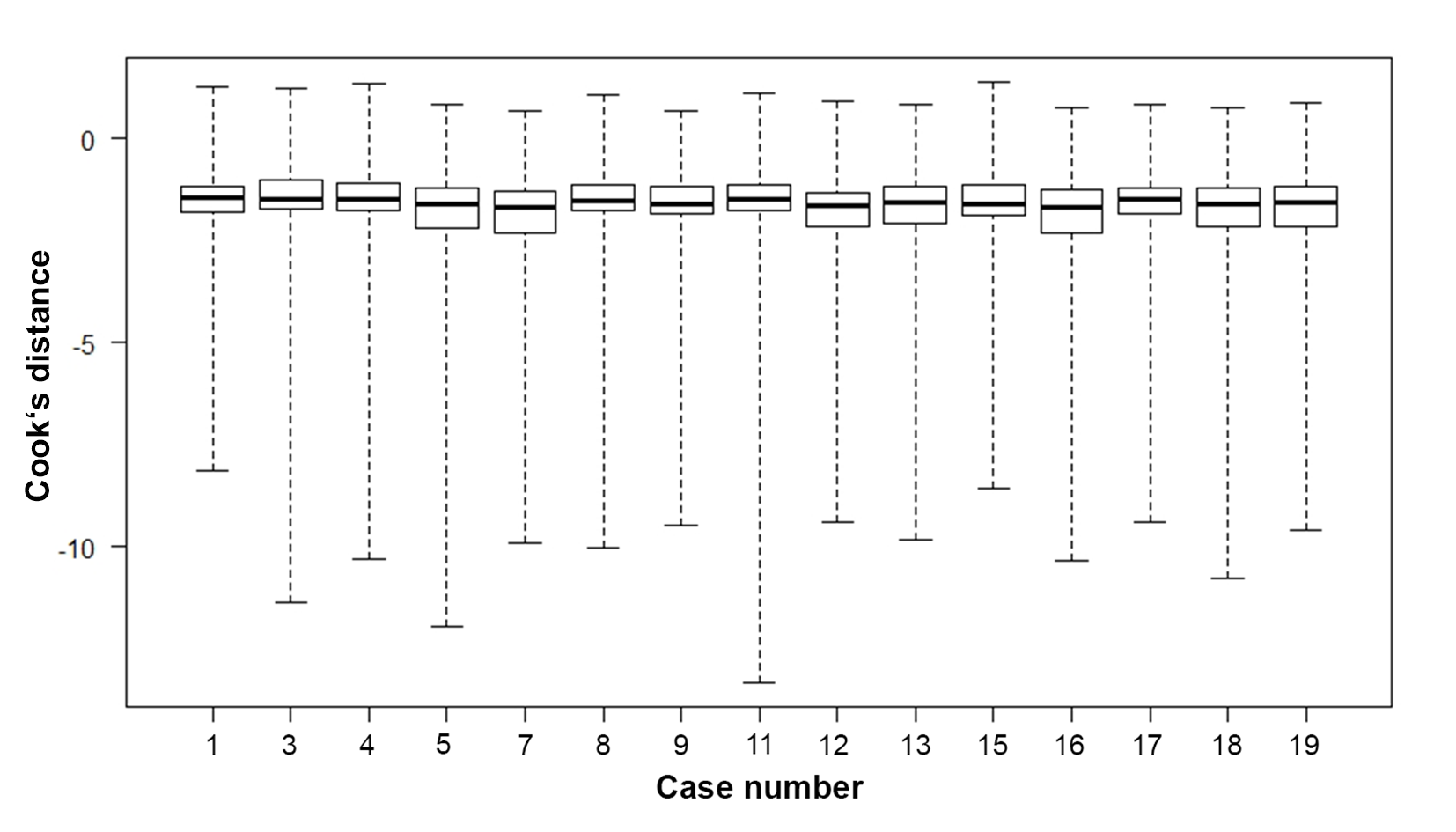

Supplement: Supplementary file 1 [file genes-12-00160-s001.zip › Figure S1_Rev1.tif]

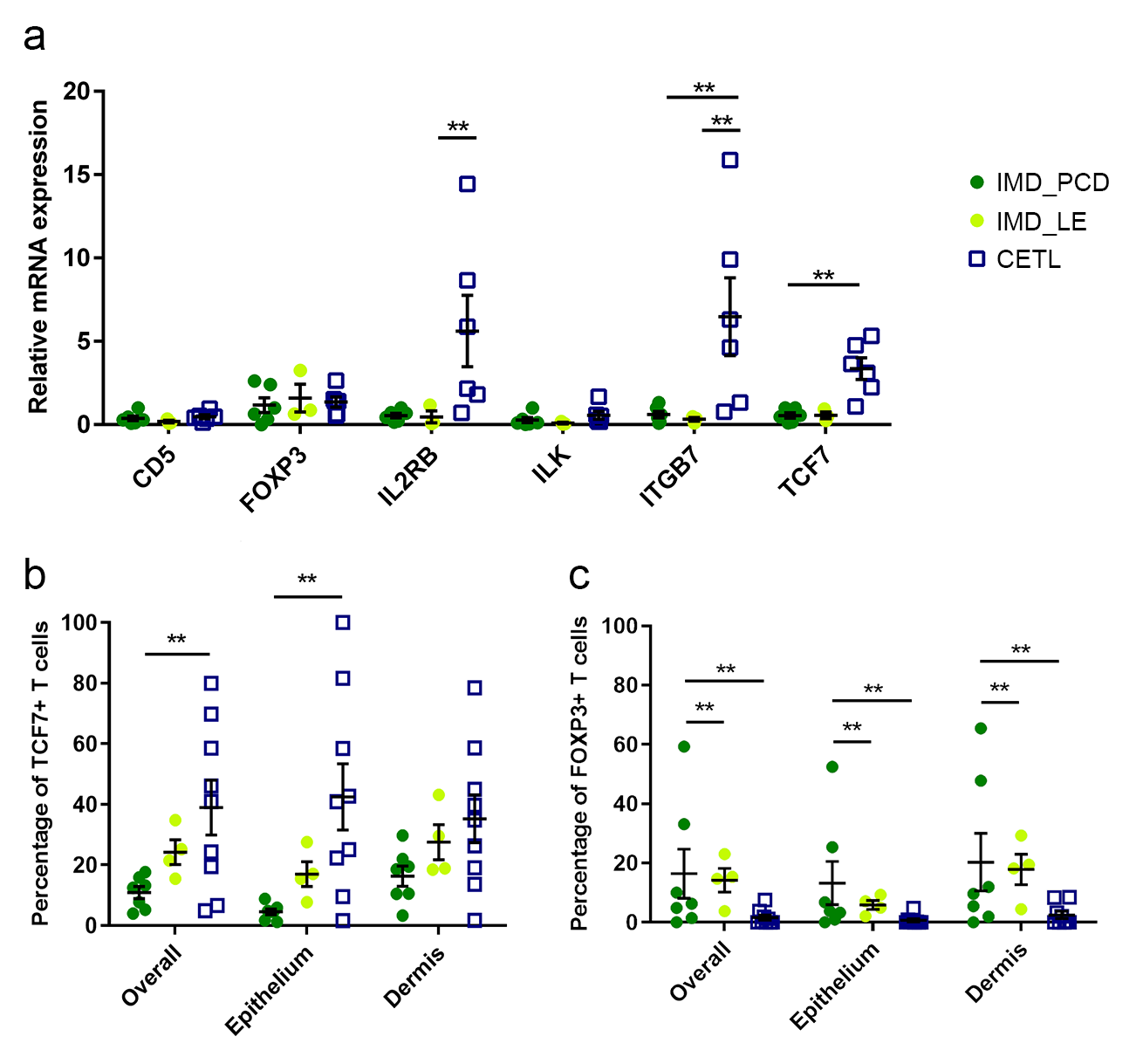

Supplement: Supplementary file 1 [file genes-12-00160-s001.zip › Figure S2_Rev.tif]

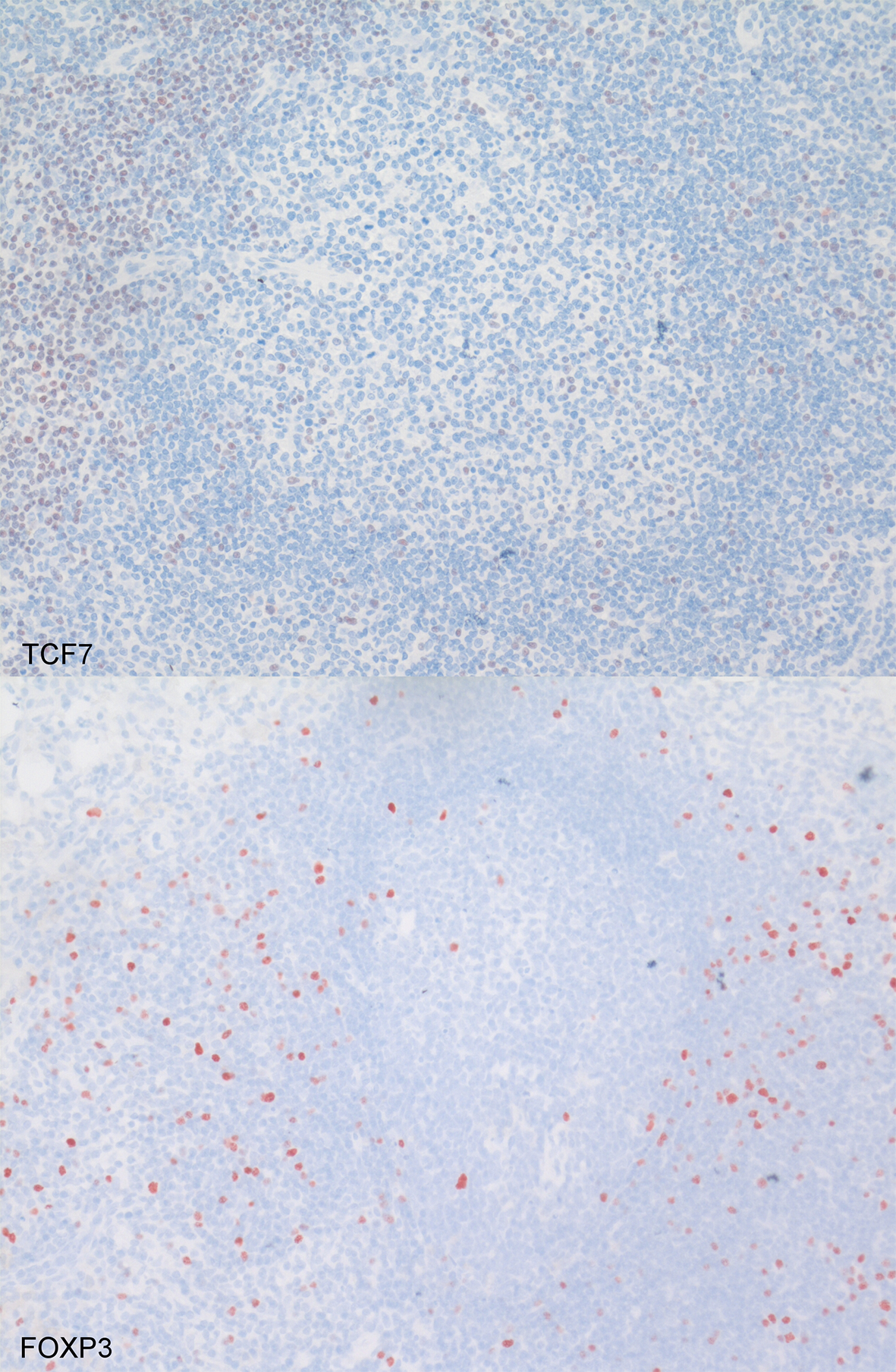

Supplement: Supplementary file 1 [file genes-12-00160-s001.zip › Figure S3_Rev.tif]
